# A rapid mixed-methods assessment of Libya’s primary care system

**DOI:** 10.1186/s12913-024-11121-w

**Published:** 2024-06-11

**Authors:** Luke N. Allen, Arian Hatefi, Mohini Kak, Christopher H. Herbst, Jacqueline Mallender, Ghassan Karem

**Affiliations:** 1 University of Oxford Centre for Global Primary Care, Oxford, UK; 2 World Bank, San Francisco, USA; 3 World Bank, Tunis, Tunisia; 4 World Bank, MENA, Riyadh, Saudi Arabia; 5 Economics By Design, London, UK; 6 PHC Institute, Tripoli, Libya

**Keywords:** Libya, Primary care, Health systems, Primary health care, Service delivery, Global health, Mixed-methods, Background

## Abstract

**Background:**

Libya has experienced decades of violent conflict that have severely disrupted health service delivery. The Government of National Unity is committed to rebuilding a resilient health system built on a platform of strong primary care.

**Aim:**

Commissioned by the government, we set out to perform a rapid assessment of the system as it stands and identify areas for improvement.

**Design and setting:**

We used a rapid applied policy explanatory-sequential mixed-methods design, working with Libyan data and Libyan policymakers, with supporting interview data from other primary care policymakers working across the Middle East and North Africa region.

**Method:**

We used the Primary Health Care Performance Initiative framework to structure our assessment. Review of policy documents and secondary analysis of WHO and World Bank survey data informed a series of targeted policymaker interviews. We used deductive framework analysis to synthesise our findings.

**Results:**

We identified 11 key documents and six key policymakers to interview. Libya has strong policy commitments to providing good quality primary care, and a high number of health staff and facilities. Access to services and trust in providers is high. However, a third of facilities are non-operational; there is a marked skew towards axillary and administrative staff; and structural challenges with financing, logistics, and standards has led to highly variable provision of care.

**Conclusion:**

In reforming the primary care system, the government should consolidate leadership, clarify governance structures and systems, and focus on setting national standards for human resources for health, facilities, stocks, and clinical care.

**Supplementary Information:**

The online version contains supplementary material available at 10.1186/s12913-024-11121-w.

## Background

Libya is an oil-rich upper-middle income country in North Africa [1]. Most of the country is desert and 90% of its 7 million people live along the Mediterranean coast [2, 3]. Life expectancy is 70 for males and 76 for females [2]. Non-communicable diseases account for four fifths of all deaths and disability-adjusted life years (DALYS) and two thirds of adults are overweight [4, 5]. Colonel Qadhafi led the country for four-decades until the 2011 a civil war which led to his removal. A series of interim governments have governed Libya over the past decade in the context of multiple competing power blocs, variably backed by overseas powers. Libya has become a major transit country for migrants seeking to reach Europe, and in 2019 migrants made up 12% of the population [6].

Perhaps surprisingly – given this context – Libya has relatively strong medical training institutions and facilities. The Ministry of Health (MoH) is increasingly focusing on the central role of primary care, and the government’s new Health Service Delivery Policy commits to “develop and organise service delivery based on the primary health care, which assures universal access, as a fundamental human right, to a health services package (defined by MOH), including emergency services at all levels of the health care.” [1]. The establishment of a well-structured primary care system also directly underpins the MoH’s ‘2030 Vision’. The Presidency Council of the Government of National Accord has also established and funded a Primary Healthcare Institute to operationalise these plans in collaboration with international partners.

In 2021, as part of World Bank Technical Assistance to the MOH, our team was commissioned by the government to perform an assessment of the primary care system. We had a three-month period to provide a holistic view of how the system currently stood and which areas should be prioritised for strengthening. In this paper we present our key findings and recommendations.

## Methods

### Approach

We performed a deductive framework analysis, using the applied policy research approach initially developed by the Social and Community Planning Research Institute [7]. Key characteristics of this method are that it is generative, dynamic, comprehensive, and accessible. We selected this approach because it is well suited for rapid analysis of policy issues [8].

**Fig. 1:**
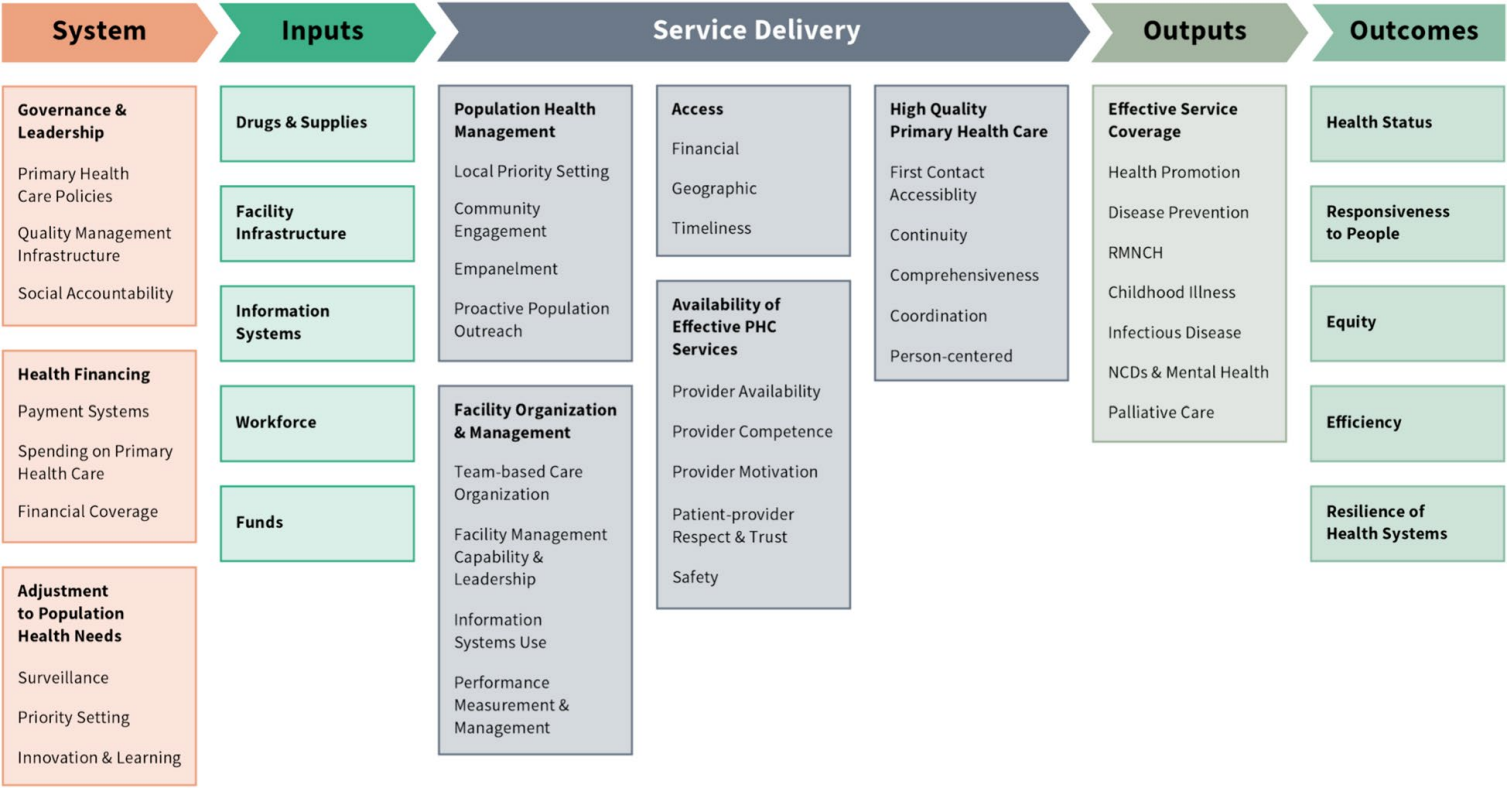
The PHCPI framework

To structure our analysis we used the *Primary Health Care Performance Initiative* conceptual framework, [9] developed by a collaborative group from the Bill and Melinda Gates Foundation, the World Bank, the World Health Organisation (WHO), Ariadne Labs, and Results for Development [10]. The framework aligns with WHO normative documents and key primary care references, [11–13] and it is specifically designed for whole-system assessments in low- and middle-income countries [10]. Our primary care system assessment focused on system components, inputs, and service delivery, as shown in Fig. 1.

We used an explanatory sequential mixed methods approach for data collection and iterative analysis, [14, 15] moving from a review of policy documents and secondary analysis of previous study findings to a short series of key informant interviews with policymakers to hone our findings and identify key recommendations.

We used an undergirding pragmatist philosophical paradigm because this approach provides researchers with broad latitude to select the research methods and techniques that best meet their needs in answering their research questions. Adherents of this paradigm hold that “truth and value can only be determined by practical application and consequences” [16, 17].

### Data collection

#### Policy documents and literature review

Our rapid search for relevant policy documents and literature was conducted in January – May 2021. This included two stages. In the first stage, we worked with the head of the national Primary Health Care Institute (PHCI) to review the library of all national health policy documents that had been published since 2011 (the data or the civil war). We performed a full-text review of all documents that mentioned primary care in the title or abstract/executive summary. We also performed full-text reviews of all national health system documents. We extracted all data pertaining to any one or more of the system, input, and service delivery domains in the PHCPI framework. We also coded and extracted all quotes that represented national commitments to primary care. Three authors (LA, JM, and AH) performed independent triplicate document review. LA performed data extraction, with a 50% sample double-checked by JM.

In the second phase, we performed a literature review using PubMed and Google Scholar, using the deliberately broad search terms; Libya$ AND (primary care OR primary health care OR PHC). We searched PubMed and the first 20 pages of Google Scholar. Title and abstract screening were used to identify papers that provided any information on the system, input, and service delivery domains in the PHCPI framework. We included empirical research studies and official reports. Opinion pieces, grey literature, and reports were included in order to maximise the data available for analysis. No date or language restrictions were applied.

We included papers and reports that covered both the public and private primary care sectors, however the government and the PHC Institute is primarily concerned with the state of the public system. Libya has committed to the Universal Health Coverage principles of providing comprehensive state-financed care to all, free at the point of use [18]. Private entities, though technically permitted to supply primary care services, are not regulated in Libya, and it is difficult to get accurate data on these providers.

After completing the literature review, we approached primary care teams at the WHO, World Bank, and Libyan government to uncover previously conducted studies, reports, and surveys that provided additional data on the primary care system. We attended international development partner meeting to obtain further data on each element of the primary care system.

In total we identified 11 key documents that contained quantitative and quantitative data assessing all aspects of the primary care system (Table 1).

**Table 1:** Documents obtained from literature review, government informants, and development partners

• 2018 World Bank patient and provider PHC survey [19] (and underlying raw data) • 2014 World Bank family health survey [20] (and underlying raw data) • 2017 WHO/MoH Service Availability and Readiness Assessment (SARA) of the public sector health facilities in Libya [21] (and underlying raw data) • 2020 MoH PHC strategy [22] • 2020 MoH & National Centre for Health Sector Reform. Well and Healthy Libya: National Health Policy, 2030 [23] • 2017 MoH. The Libyan Health System: Study of Medical and Allied Health Education and Training Institutions [24] • 2020 National Centre for Health Sector Reforms and WHO. Reorganized Structure of the Ministry of Health [25] • 2019 World Bank Libya Local Governance Forum. Service Delivery in the Perspective of the Health Sector in Libya [26] • 2021 WHO Libya health sector bulletin [27] • 2015 WHO PHC country profile and vital signs [28] • 2020 WHO Libya Country Office. Libya Annual Report 2020 [29]	

### Secondary analysis

We followed the key stages of deductive framework analysis to extract, sort, and analyse the data; 1) familiarization; 2) indexing—annotating each source to link data with relevant domains from the PHCPI framework; 3) charting—‘lifting data from their original context and rearranging according to thematic area’; and 4) mapping and interpretation of the dataset as a whole [7].

The key documents included a mix of quantitative and qualitative reports. Working with the WHO, World Bank, and MOH, we were able to obtain the underlying raw data for service readiness, patient and provider survey feedback, and family health survey data. We performed simple summary statistics in MS Excel to generate national summaries aligned with each of the PHCPI domains.

The Libyan political context has presented long-standing challenges to rigorous data collection. Among available secondary sources (i.e. those listed in Table 1), three contained underlying raw data. Two surveys were conducted by the World Bank as part of its long-standing technical assistance program, and the third, the Service Availability and Readiness Assessment, is a WHO-developed standardized assessment tool that affords comparison across and within countries.

### Moving from document review and secondary data analysis to qualitative interviews

We produced a first draft of the national assessment, grouping all data under each PHCPI domain and noting areas of agreement between data sources, dissonance, and silence. To extend our understanding, triangulate emerging findings, resolve dissonance, and fill in knowledge gaps, we conducted a targeted set of semi-structured interviews with a sample of key informants holding senior leadership positions within Libya’s primary care system.

Our topic guide (Appendix) focused on key areas of uncertainty within each PHCPI domain. We also sought to gather first-hand experience of delivering PHC services in contemporary Libya; perceptions of the main challenges faced by patients and providers; current efforts to advance PHC; and priority areas for reform.

We used purposive sampling and snowballing. Given that we had to perform the document review, generate initial findings, identify interviewees, perform interviews and analyse all data within 12 weeks, we were only able to interview a very small group of people. As such, we focused on system leaders with broad experience of primary care in Libya. Working with the lead of the national PHC institute, we performed a stakeholder analysis to identify the major organisations contributing to the development and delivery of primary care in Libya. We identified the seniormost representatives of these organisations and invited them to participate via email. Given that there are very few people working directly on primary care policy in Libya, we asked our interviewees to recommend further people to interview. In total, six interviews were conducted in Spring 2021 by LA and JM with senior policymakers working in the national GP Society and Libyan GP training scheme, the National Centre for Disease Control family practice team, the WHO Libya Country Office, and the WHO Eastern Mediterranean Regional Office primary care team. All interviews were conducted via Microsoft Teams in English and lasted 45–60 minutes. All participants spoke fluent English. Notes were taken during the interviews. We used thematic analysis [30] to code the interview transcripts and identify the main themes.

Our interviews were low risk, and the power imbalance favoured our interviewees, all of whom were high-level policymakers. Interviewees were fully informed of the project scope and provided consent to participate. We have kept responses anonymous to protect them from any potential risks. The recorded interviews and interview notes were stored on a secure, password protected folder within the EU. These data will be destroyed after seven years.

### Synthesis and interpretation

We iteratively repeated the deductive framework analysis stages to map and analyse the totality of data with reference to the PHCPI conceptual framework. After every interview, each domain was updated, with new themes and findings being used to update the topic guide for the subsequent interview.

In writing up our report, we presented our findings narratively by thematic area, following the structure of the PHCPI framework.

### Reflexivity

Interviews were conducted by LA – a white male British family physician and global health policy expert, and MK, a female Asian health system specialist. Both researchers have extensive experience working with primary care systems in high-, middle-, and low-income settings, but no prior experience working in Libya’s clinical system. LA and MK worked in equal partnership with the wider research team that was comprised of World Bank middle east health specialists, JM a senior British health economist, and GK, a senior Libyan primary care policymaker with clinical experience working as a Libyan primary care doctor.

## Findings

### System

#### Governance and leadership

The public primary care system is the first of three levels of care. The Municipality (*Baladya*) manages the primary care facilities, while a secretariat of health at the district level manages the hospitals, including specialized hospitals. Libya refers to primary care as ‘Primary Health Care’, and for the remainder of this report we will use the term PHC. There are three types of PHC facility that provide comprehensive primary care services, ranging from small PHC units to large polyclinics. “communicable disease centers” also operate within the PHC tier but offer vertically integrated, disease-specific services.

Libya has a number of well-developed primary care policy documents and institutions, but thus far they have not yet translated into tangible action. For instance, the government acknowledges that the provision of health care as a basic right [23]. It has developed a 2020–2022 PHC strategy, signed by the Minister of Health in September 2019, its 2030 vision emphasises the centrality of PHC, and the country has invested in the creation of the PHC Institute. However, these documents and institutions have not yet led to marked improvements in the quality of services. The government does not have a quality-of-care framework, and it has yet to clearly outline a basic package of services.

In spite of the headline principles that are in place, there is a paucity of operational guidance. PHC governance remains fragmented, with multiple institutions sharing overlapping responsibilities for various aspects of PHC delivery. The result is that each institution tends to view these responsibilities as belonging primarily to other agencies. Compounding this, policy-making organisations do not routinely work together. Interviewees felt that senior politicians typically perceive PHC as intangible and strategically unimportant, especially in comparison to secondary care, which often appears medically more urgent, making it is easier to make the case for creating tangible prestigious services and thereby win political capital. There is a need for the PHCI to take a greater lead in developing a long-term strategy around human resources, service delivery models, and financing.

*“The current system is very fragmented with no clear leadership” –Key informant interviewee*

The fragmentation of PHC governance has impeded the ability of local facilities to respond to the unique needs of their local communities. Interviewees, policy documents, and a number of recent national PHC workshops co-hosted by the WHO all emphasise the desirability of devolving administrative and financial authority to local levels, while mitigating potential risks. The current model is seen as fractured, restrictive, inefficient, and inadequately funded, with a lack of guidance on standards and non-transparent flows of governance and accountability. According to the 2030 National Health Policy, there is no consistency in the package of services offered at any of the facility types, nor in the structure, staffing, or resources. There is no established national quality management infrastructure, despite the fact that highly variable PHC quality remains a major weakness of the system. Two interviewees noted that work to establish quality indicators and an essential basket of services is underway. The National Centre for Health Sector Reforms has argued that the mistaken pursuit of localized autonomy has aggravated the problem of variability and led to the undesirable emergence of a number of “power centres” and “budget centres,” which has weakened the authority of the ministry of health to oversee and administer the health system [25].

The current system does not involve patients or communities in decision-making processes. Community-level involvement is a core pillar of the Alma-Ata [11] and Astana Vision of Primary Health Care [31]. But the imperative to provide basic services in extremely challenging conditions has meant that investing in community engagement has fallen as a priority. Various policy documents intimate that community engagement should play a larger role in shaping national and local services, but there is no explicit strategy for achieving this stated objective. There are currently no routine mechanisms for seeking, collating, analysing, or acting on patient and community feedback on their experience with care. One-off surveys have been conducted with international partners and research institutions, but these activities are not yet built into routine practice.

#### National health financing

In comparison to secondary care PHC is chronically underfunded. As with most countries around the world—including those with strong PHC systems—secondary care tends to capture a disproportionate share of health spending [5, 32]. Whilst the health system needs major investment, in 2017 the actual capital expenditure on the health sector was 10 percent of the budgeted expenditure, meaning that 90% of the funds allocated to health were not spent. Salaries are late or unpaid for a large proportion of public workers according to the government’s accounts, including 81 percent of health staff [23]. This is a major pain point for the PHC system.

Libya’s fragmented budget system makes it difficult to ensure that resources are aligned and available to meet the needs of patients. The health budget is divided into four chapters, and the centralisation of staffing, capital expenditure, and medical supplies—handled by three different non-health-related ministries—leads to delays and inefficiencies. The 2020–2022 PHC strategy recommends delegating a degree of financial autonomy to PHC units, as well as moving to a capitation payment system, starting with recurrent costs and then extending to cover the entire cost structure of PHC centres, with the aim of covering the provision of the essential package of health services.

In line with WHO recommendations, almost all care is provided free at the point of use, but the costs of medicines and supplies present a barrier for poorer patients. In 2018 the World Bank conducted a patient survey with over 1,000 patients and over 500 service providers. This well-conducted project found that 97 percent of patients were not charged fees for accessing PHC services – including ‘under the table’ payments. However, 51 percent of them considered the costs of medical services in Libya to be a problem, and 13 percent forwent recommended PHC services or medicines because of cost concerns. Costs are associated with medicines, supplies, equipment, and diagnostics. Where local services are deemed to be inadequate, patients commonly look to the private sector, which incurs additional costs for accessing care. In 2011, out-of-pocket expenditure accounted for 36 percent of total health spending [33].

#### Adjustment to population health needs

Libyan PHC policy documents recognise that services should be responsive to population health needs. However, provision varies widely among facilities, with no particular logic underpinning the distribution of specialist services. Epidemiological data are recorded by a national Health Information System that collates facility-level data from paper records, and by the National Centre for Disease Control, which gathers and reports epidemiological data. Yet these systems are not used to shape PHC service delivery. The absence of a national electronic health records system makes data collection and analysis onerous.

The focus of many local facilities is on maintaining basic services. The current PHC system does have a culture of innovation and learning that is required to continually adapt and improve, based on changing population needs. Simply maintaining the status quo is perceived as already very challenging. Many health care clinical staff simply lack the time, training or headspace to engage with continuous improvement.

*“It is such a challenging environment there is not time or space to think about innovation and learning”-Key informant interviewee*

### Inputs

#### Drugs and supplies

**Fig. 2:**
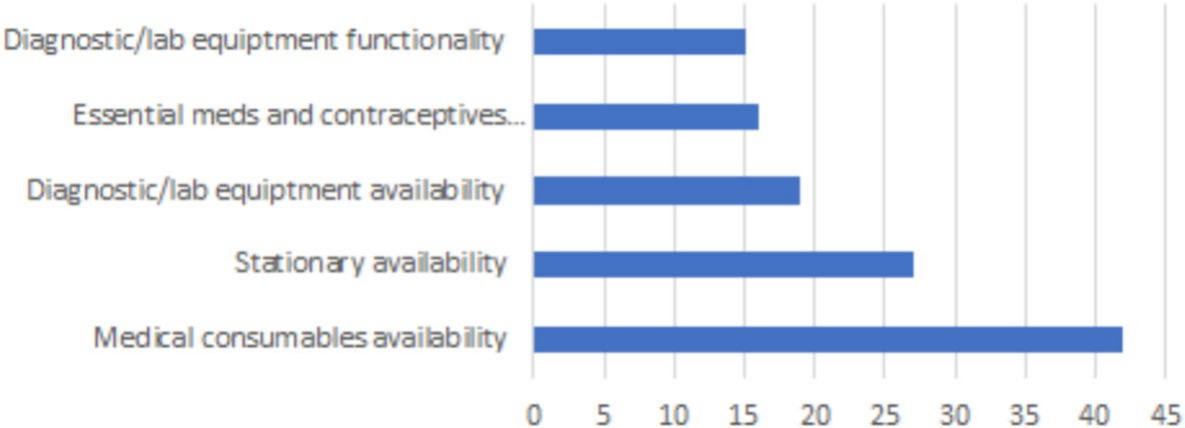
Percentage of staff reporting issues with supplies Source: World Bank provider survey 2018

Logistical issues with drugs and supplies are two major challenges for the PHC system (Fig. 2). According to the latest Service Availability and Readiness report, the mean availability of essential medicines was 10 percent in 2017 [21]. These WHO assessments use serial standardised reviews with a representative number of facilities across the country. They are generally well conducted, however the report we had access to did not provide any information about the specific methods employed in Libya, making critical appraisal difficult.

The World Bank has conducted the only patient and provider survey in Libya since 2011, as far as we are aware. The questions are well-worded, and were asked by localy-recruited data colelctors in participant’s own language. The sampling strategy was rigorous and the sample size enables national generalisations. The survey suggests that this issue of low medicine availability is perceived by both clinicians and patients as one of the biggest problems facing PHC facilities (Fig. 5). The lack of sufficient supplies is a very common reason given by patients for bypassing their nearest PHC facility when seeking care. The absence of an essential package of services means that core medicines are often not available.

#### Facility infrastructure

**Fig. 3:**
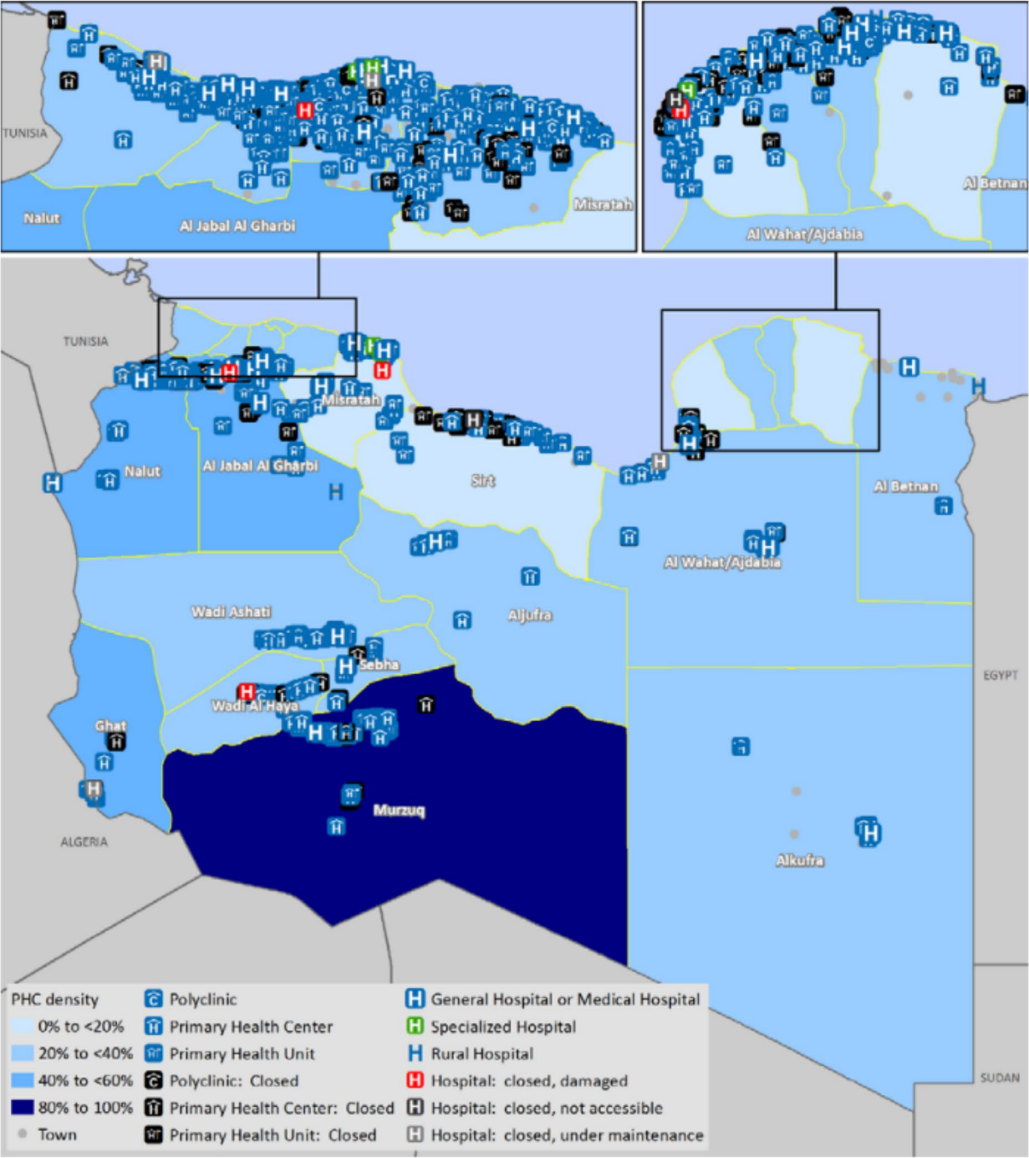
Distribution of PHC facilities Source: 2017 SARA report

Libya has an adequate density of facilities overall, albeit with a skewed distribution across the regions (Fig. 3). The stated health facility density of 2.8 health facilities/10,000 people is above the WHO target of 2/10,000, but this count includes private facilities. There are no national standards for facility infrastructure or facility density, which means that the distribution and physical status varies widely.

According to government records, there are currently 728 PHC Units, 571 PHC Centres, and 56 Polyclinics. Units provide maternal, neonatal, nutritional, child and school health services, vaccination, early diagnosis of infectious diseases, health promotion, registration and follow up of chronic diseases, curative services, local water quality monitoring and assessment of local environmental risk factors. PHC Centres offer supervision for PHC Units, the same basket of services, plus dental care. Polyclinics offer more specialised care for catchment areas of 50–60,000 people, accepting both walk-ins and referrals from PHC Units and Centres.

Although Libya has 1,355 PHC facilities in all (according to official figures in the latest Health Sector Bulletin) [27], 273 are closed because of either a lack of maintenance (51 percent), inaccessibility on account of conflict (20 percent), physical damage (19 percent), or other parties occupying it (11 percent). According to the 2017 Service Availability and Readiness Assessment (SARA) report, only one-third of primary healthcare clinics are fully functional and only 40 percent offer basic maternal and childcare. The mean basic service provision index is 45 percent [21].

#### Information systems

Libya has a nascent health information system that would benefit from deeper investment. There are more than 400 civil registration offices, and Libya has an automated vital registration system [34] however medical records are still paper-based and health management systems are not in routine use at the national or local level [29]. Existing health records systems are not interoperable across the wider health network. This can frustrate data-driven central planning and coordinated patient care. A District Health Information System (DHIS-2) is currently being implemented but interviewees raised concerns that it is currently too far removed from clinical care to offer meaningful patient benefit. A health information system (HIS) workplan is currently under development.

*“The new system [DHIS-2] is not being designed with clinical users in mind… It will not be useful for them”-Key informant*

#### Workforce

Libya has a large health workforce, but the skill mix is unbalanced. The cumulative density of physicians, nurses, and midwifes is 8.68/1,000, which is virtually double the ratio the WHO recommends for achieving universal health coverage [23]. However, there is a surfeit of axillary clinical staff and a shortage of nurses, general doctors, and family physicians. There is a drive toward boosting the number of family physicians—currently around 124—and experiments with introducing community health workers to operate in a sensitization and signposting role, directing people to appropriate PHC services. The government is also keen to train more generalist nurses for primary care, moving away from a recent trend to train single disease specialists.

PHC facilities have no autonomy in changing their personnel structure. There are no national staffing standards for primary care, and the number of staff attached to each primary care facility ranges from two to several hundred, and more than 1,000 for large polyclinics, according to the latest SARA report [21]. Workers are not distributed across the country equitably, and there are concerns that competencies are variable. The average total staff per PCH facility is 88, and many staff members only provide services for single health conditions such as tuberculosis. In some facilities up to 50 percent of staff are dedicated to administration functions [21]. Furthermore, there are a large number of paid “ghost workers” who appear on facility books but do not show up or perform any work.

There are also 302 facilities that currently do not offer any services at all, yet collectively employ 14,500 health workers [21]. Many more facilities have hundreds of staff on their books but offer very limited services. These staffing figures suggest that the limited money that is being spent on PHC could be allocated much more efficiently; employing fewer but more highly-skilled clinicians, retraining ‘vertical’ single-condition workers to address a range of common primary care conditions, and ceasing all payments to ghost staff and non-operational facilities.

#### Facility funds

Facilities do not have a high degree of control over their funds. This constrains managers’ abilities to shape services to meet local needs. Facilities are able to manage non-wage recurrent expenditure (under Chapter II of the national budget), but staff salaries, capital expenditure, and medical supplies are all financed centrally by three different departments.

There are no national data on the proportion of funds that are actually received under each chapter each year, or how these funds are spent. PHC facilities do not receive adequate finances to stock medications and equipment. In a robust survey of 541 health care workers, 43 percent had not received their salary in full over the previous three months, and 73 percent had not been paid on-time [19]. The inability to manage funds makes it hard to retain staff and build trust with the local community. Interviewees felt that issues with funding, salaries, and procuring appropriate supplies had deep roots and complex antecedents at the national, regional, and local levels.

*“Late payments are not something that can be easily fixed. It affects the whole public sector.”-Key informant*

### Service delivery

#### Population health management

The current PHC system lacks established processes to identify systemic variation and use this knowledge to develop new actions to improve population health. The over-centralized control of purchasing and service design not only impedes local priority setting but also undermines the case for deepening community engagement and for translating national policies into local strategic action plans that go beyond delivering a universal package of basic services. Empanelment has not been introduced, reducing the incentive to employ proactive outreach to improve population health outcomes in local communities. However, there are a number of successful initiatives to care for marginalized and rural populations, such as mobile outreach clinics that provide primary care services.

#### Facility organization and management

PHC facilities could be more effectively organized by providing managers with the training and administrative authority to use their data and staff to monitor and continually improve care quality. According to key stakeholder interviewees, the competence of facility managers ranges widely, and there is no culture of improvement and innovation. There is a general sense that managers should be given better training and more autonomy in running facilities.

Health intelligence is not routinely gathered or used at the facility level. Care is delivered by multidisciplinary teams, but there is wide variation in team composition, responsibilities, and competencies. Many facilities lack adequate supplies of medicines, supplies, and medical equipment, and staffing can be patchy and unreliable. At the start of the COVID-19 pandemic, up to 90 percent of PHC facilities closed because they lacked personal protective equipment (PPE) [29]. Performance measurement and management systems are in place, but are highly variable.

#### Access

**Fig. 4:**
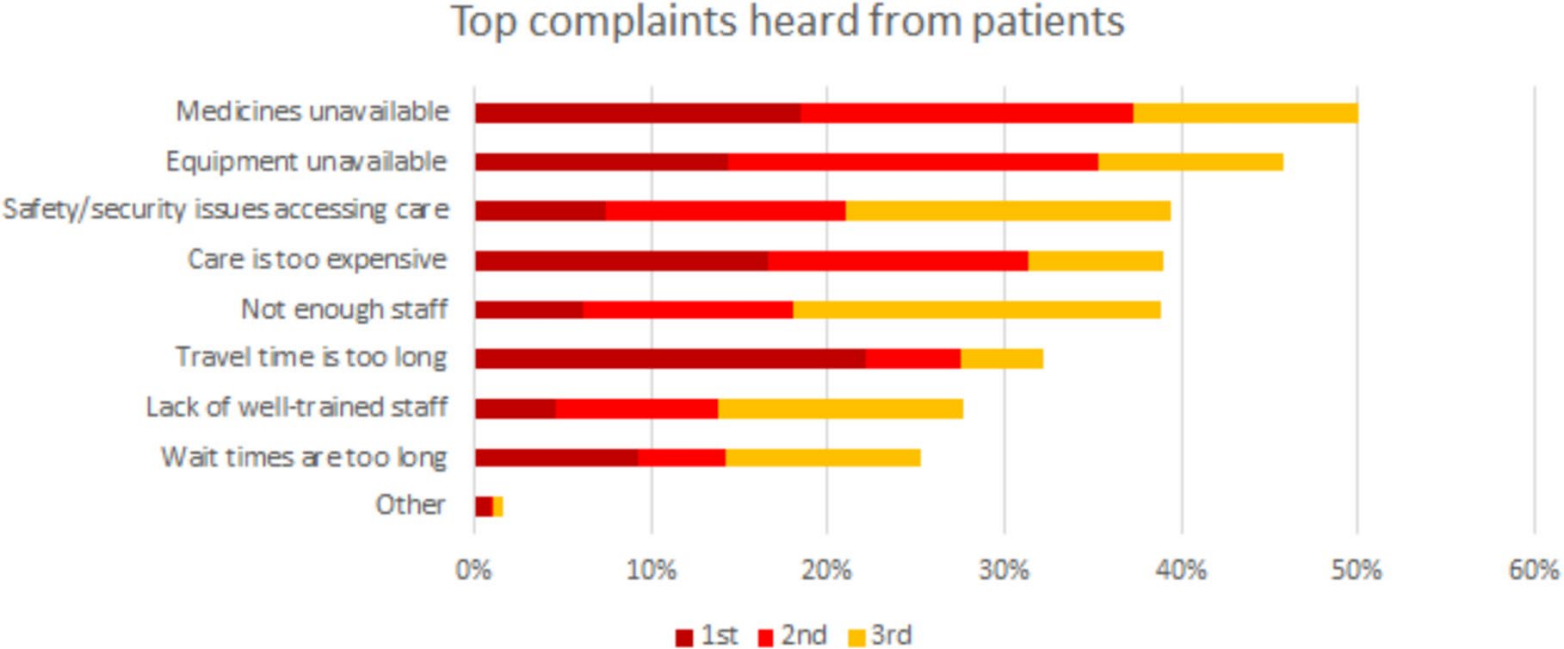
Top complaints raised by patients about PHC facilities Source: World Bank 2018 patient survey

Financial access to services appears to be good but does not reflect widespread perceptions of low service quality. As stated above, some 97 percent of patients state that they are not charged to access PHC services [20], however clinics often do not have medicines or supplies so patients have to buy their own on the private market. As such, financial considerations represent an important barrier to access (Fig. 4). The health ministry feels that perceptions of low quality lead some to opt to pay for private sector services [23] and one-fifth of patients stated that they forwent medical care in the preceding year because of cost in the preceding year because of cost in the World Bank’s 2018 survey [20]. There are no up-to-date PHC-specific health account data on health spending, and there are no available data on impoverishment or catastrophic expenditures.

**Fig. 5:**
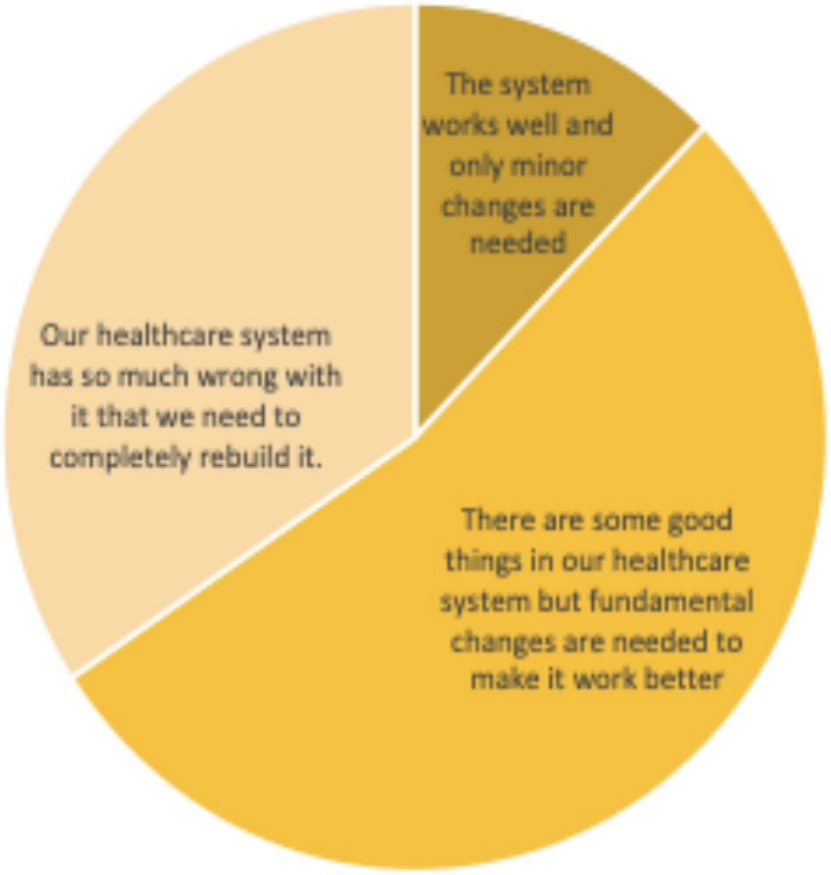
Patients’ views of the PHC system Source: 2018 World Bank Patient Survey

Despite extremely challenging conditions, Libya has managed to provide its citizens with good geographic access to free PHC services, according to the 2018 World Bank survey: more than 90 percent of patients reach their chosen clinic within 30 minutes of travel time [20]. A quarter of patients bypass their nearest clinic typically because of concerns that medicines or equipment will not be available [20]. Conflict and political volatility pose grave external threats to sustained, coherent PHC service delivery and governance. More than 60 percent of patients felt that they received high-quality care. Nevertheless, the same group of patients felt that fundamental changes were needed to make the system work better (Fig. 5).

Wait times are generally low, but opening hours can be unpredictable, and PHC is not available 24/7. The majority of PHC facilities use a combination of appointment booking for chronic or routine conditions, and walk-in systems for acute or urgent care. In practice, however, patients tend to show up and wait for services without an appointment. Although this can lead to queues at busy times, the median wait time is in fact only 15 minutes [20]. Most facilities publicise their opening times, but they are not always actually open during these times.

#### Availability of effective PHC services

Although patients can access facilities fairly easily, there is a systemic issue with the availability of adequate staff, supplies, and services. Although there are high numbers of staff working in the health sector, there is a relative shortage of doctors (especially family physicians) and nurses. Stockouts are another major issue. Security issues and electricity blackouts present further barriers to effective health care services. One of the main reasons given for bypassing certain PHC facilities in favour of others, or in favour of private care, is that some facilities do not offer the services the patients need. One interviewee noted that availability is not a major issue for many patients because they know which facilities function well and which ones do not, and simply bypass the poor ones. While this may be true for some urban patients, it can be more problematic in rural areas where the next clinic could be a long distance away.

At universities in Libya, staff receive a reasonably high standard of initial medical and other health-related training. By contrast, there is a widespread feeling that, beyond university, in-service training is insufficient to maintain high-quality care. There is no agreement as to the job description of doctors, nurses and other PHC-providing clinical team members. This makes it hard to guarantee that a trained care provider with the requisite skills will be present. Clinicians are generally eager for further training so that they can stay up to date, but opportunities are sparse. Although the vast majority of staff and patients perceive clinicians to be competent and well-trained, 12 percent of patients stated that their nearest facility does not have qualified or knowledgeable clinical staff. There is no national data on the minimum objective clinical competency of staff. Patient safety data does not seem to be routinely collected or used, nor is there routine use of a human resource management system to keep track of staff placements, training, and promotions to manage staff deployments and growth.

Most PHC facilities do not have a family physician. Family medicine residency training is unpaid and trainees have to come in to do learning sets on their days off to avoid clashes with other clinical commitments. The fact that the residents are willing to persist despite these obstacles speaks very highly of provider motivation and the quality of the training on offer. Nevertheless, this arrangement is also a barrier to training the 7,000 additional family physicians the country needs, according to the director of family physician training.

#### High-quality primary health care

PHC is the “first point of contact” with the health system by default rather than by design. Because of the large number of PHC facilities, it is often more convenient to visit a clinician at a PHC unit rather than a hospital. The problem is that, despite this central role, PHC does not perform a gate-keeping function, and the current system does not make any provisions for this role. Libya’s endorsement of the Declaration of Astana [12] and the WHO PHC operational framework [35] implies that moving toward this role remains an aspirational ideal for the PHC system, but it is not an explicit policy-level goal. Patients are able to present to virtually any public or private facility and any level of care without referral or the need for registration. This is appropriate for the current system, because imposing a rigid referral system would be counterproductive. But there is a well-founded aspiration to move toward a more integrated system.

Continuity of care is weak: patients often experience their care as a series of disjointed and isolated interactions because the lack of empanelment and named clinicians hampers relational continuity; the non-systematic use of interoperable medical records hampers informational continuity; and weak care coordination and two-way communication between specialities hampers management continuity.

Comprehensiveness is mixed. A wide range of services are offered in the PHC sector, but poor planning means that there are large gaps and overlaps in the services offered within each community. There is no guidance on which services should be provided. However, a package of essential PHC services is in development. General facility readiness is 45 percent according to the 2017 SARA report [21].

Coordination is undermined by the current PHC model. Clinics are not responsible for a geographically-defined population of patients, and patients do not have a clinician who is responsible for coordinating their care. More generally, PHC does not play a coordinating role across the course of treatment and across sites of care. The unavailability of interoperable and unified patient records is a further impediment; and currently, secondary care is not expected to inform PHC teams of what they are doing for patients referred to them.

“Whole-person care” is very slowly supplanting disease-oriented care. The vast majority of doctors staffing PHC facilities are secondary care clinicians, steeped in the disease-oriented, post-preventative, biomedical model. Family physicians are taught the biopsychosocial approach to patient care and are gradually advancing the culture of shared decision-making with patients. However, at the time of writing, there were only 134 family physicians in the country.

#### Summary and policy recommendations

Service quality is highly variable, partly driven by the lack of an established package of essential health services and standards. Introducing an essential service package should help to raise quality by focusing procurement and logistical efforts on the core set of medicines and supplies required to offer the basic range of primary care services in every community. Similarly, introducing quality standards for essential staffing lists, job descriptions, and facility manager training standards is the right starting point in moving toward the reduction of unwanted variation in service quality.

Fragmented centralisation characterises and undermines the Libyan PHC system. PHC facilities do not have the financial or administrative authority to organise local staffing, supplies or services. Central government ministries are poorly coordinated and often lack adequate technical capacity, which can lead to gross inefficiencies in the distribution and deployment of PHC resources. This impacts comprehensiveness and service quality. The new PHC Institute could play a key role in coordinating the disparate government agencies to match supplies and staff to local population needs.

The absence of interoperable electronic health records is a critical weakness. Health surveillance, priority setting, performance management, and the tailoring of services to meet local needs are all possible with traditional paper records, but it is much less efficient than electronic records. The introduction of DHIS-2 is welcome, but it needs to engage more with clinicians during the piloting stage. Besides boosting health surveillance and planning, a move to electronic patient records could also advance coordination and continuity of care across primary and secondary care settings.

Sustained high-level political buy in is essential for the future of PHC. The current very modest budgetary allocation to PHC points to the fact that the value of primary care is not fully understood at the highest levels of government. Making greater political investment in reforming the multidisciplinary agencies that impact primary care funding, procurement, staffing, and operations will not be easy, but this work is needed if the wider systemic problems that underlie the critical issues in the health sector are to be fixed. Breaking up the ambitious goal of PHC reform into a series of more manageable, shorter-term, “quick win” modules may help by giving politicians tangible and attainable legacy projects. Consolidating PHC policy leadership is also required to rationalize the disparate flows of governance and accountability.

The nascent culture of family medicine needs to be nurtured. First-class PHC is built on first-contact, continuous, coordinated, comprehensive, and person-centred services. These are the core principles of family medicine that are being advanced by the growing cadre of family doctors. The government should incentivize training with the aim of staffing every PHC facility with at least one family physician. The introduction of the gatekeeper role, empanelment, and interoperable medical records will further support the realization of high-quality primary health care, but patients are likely to resist any attempt to limit their choices until they perceive that their local facility is able to offer an adequate level of care.

The PHC workforce should be reoriented toward skilled generalists. The current workforce is disproportionately skewed toward administrators, low-level clinical assistants, and nurses trained to manage single diseases. Improved efficiency and governance structures are required to tilt the balance toward a smaller but more competent and broadly skilled workforce. Existing standalone communicable disease facilities and workers should be retrained and redeployed to provide a broader range of services, aligned with emerging health challenges such as non-communicable diseases and mental health problems.

COVID-19 has brought into sharp focus the potential role of PHC in providing comprehensive services across the spectrum—from outreach, prevention, and screening to diagnosis, treatment, and rehabilitation. Although the pandemic has crippled many facilities, it has also drawn attention to the importance of adequately resourcing the first level of the health system to enable it to provide comprehensive services.

## Discussion

As far as we are aware, this is the first holistic assessment of Libya’s primary care system. We found that the Libyan PHC system has a number of strengths. There is a high level of national policy commitment to universal health coverage, equitable service provision, and the development of a strong PHC network. There are a large number of PHC facilities, and a reasonable number of staff providing relatively good levels of geographic coverage. Physical and financial access to services is good, with short travel times and free care at the point of use. Staff tend to be highly committed to their work, and patient-provider respect and trust are high, according to the most recent surveys. Recognising the importance of family medicine, a small but growing number of doctors are being trained in this specialty.

However, broken financing, staffing, and procurement systems severely hamper quality. There is wide variation in the quality of care, the comprehensiveness of services on offer, and general service readiness. This is partly driven by the absence of national standards that spell out the staffing and other resources that ought to be available at every facility if they are to deliver a set list of essential medical services. Patients are savvy about which facilities function better and tend to bypass those that lack the requisite staff, medicines, or access to investigations. Routine mechanisms should be introduced to collect patient and community feedback on experiences of care.

The complex tension between centralisation and devolution requires careful unpacking. There are many proponents of the policy perspective that PHC managers should be vested with a higher degree of responsibility for staffing, procurement of medical supplies, and the design and delivery of services. However, a high degree of training, experience, professional integrity, and competence is required to undertake this complex responsibility well. Efforts to upskill the PHC manager workforce should be complemented by an effort to reform the currently centralized staffing and procurement systems with the aim of providing the resources needed to consistently deliver a basic package of services at every facility. PHC stakeholders also need to collectively agree on the best way forward, looking to the strengths and weaknesses of both approaches, and looking to learn from other countries.

Providing high-quality PHC will be difficult without the introduction of electronic health records, empanelment, and upscaling of the GP training scheme. In Libya, doctors do not currently perform the care coordination role, and PHC is not the first point of access to the health system. Care coordination and two-way referrals between primary and secondary care, and between different team members, are predicated on the existence of shared medical records. It is possible to send paper records with a patient, but interoperable medical records make this much easier. Empanelment connects patients with a given PHC facility and its clinical team. This gives clinicians greater ownership over patient care and forms the basis of capitation payment systems that can incentivise proactive health promotion and community engagement. The current PHC workforce is mainly comprised of axillary clinical staff and secondary care-trained doctors. There is a need for more nurses and doctors trained specifically in primary care, including patient coordination across the health system.

There is a widespread appetite for reform among PHC stakeholders, but a number of broader contextual factors pose a major threat. There is remarkable consensus among the various Libyan PHC agencies and NGOs that the system needs a wide range of core reforms. But ongoing conflict, political upheaval, and rapid personnel changes at the higher levels of government have made it extremely challenging to work toward long-term reform with any meaningful consistency. A strong civil society/patient voice calling for PHC reform does not currently exist, and recent budgetary discussions barely mentioned primary care.

The PHC community may be able to make incremental reforms and use pilot sites to test core innovations. Communicating the value of topflight primary care in a way that is digestible to the public and politicians—and the armed groups that pose a threat to PHC facilities, patient, and staff—may help to carve out the essential policy space for this work. The growing burden of noncommunicable diseases is widely perceived as a strategic threat to national health and wellbeing. These chronic diseases are best managed through primary care, and growing recognition of their significance should help spur deeper investment in PHC and the reforms required for continuous and coordinated management of long-term conditions.

This study has a number of strengths: our multidisciplinary team comprised of Libyans and international primary care system experts conducted careful search for all relevant policy documents and quantitative data. We did not perform a full systematic review because we lacked the time, however in partnering with national academic and policy leaders we are confident that we did not miss any important studies or Libyan policy documents from the past decade. We employed the widely used PHCPI framework to ground our deductive analysis, and our exploratory mixed-methods approach enabled us to get the most out of our interviews. Given a longer than three months, we would have interviewed a wider range of Libyan care providers, however there was a very high level of agreement amongst our interviewees and we felt that we achieved data saturation. Ideally, we would have interviewed patients, however this was not logistically possible at the time. Our findings are geared for policymakers and will inform the next phase of national reform. Whilst this assessment brings together multiple evidence streams, our understanding of Libya’s PHC system is still incomplete. Many of the underlying surveys are over five years old, and much has changed during the course of the pandemic. We have very little empirical data on community engagement, the use of surveillance data, priority setting, population outreach, models of care, out of pocket payments, information system use, and adherence to quality standards. Other important limitations are centred around the paucity of data on the Libyan primary care system. Besides the 2017 SARA report and the 2018 World Bank survey, there is very little high quality empirical evidence on how the system is actually functioning across the country. We found major data gaps around the core aspects of high-quality primary care delivery (continuity, coordination, comprehensiveness etc.), an absence of subnational data, and a lack of interval data meaning that we are unable to comment on temporal trends. As such, we had to rely on our key informants who are very well placed to provide a broad overview of the system. However, better data are foundational to future attempts to reform the system.

## Conclusion

Libya has a high number of staff and a high density of primary care facilities. Years of conflict have fundamentally broken the governance, financing, and logistics systems. The PHC Institute is well positioned to begin rationalising and consolidating PHC resourcing and governance, and should start with establishing basic standards.

### Supplementary Information

Supplementary Material 1.

## Data Availability

All underlying data are publicly available (Table 1) except for the interview transcripts. These transcripts will not be made available in order to protect participant confidentiality. For data requests please contact Dr Luke Allen, corresponding author at: luke.allen@lshtm.ac.uk.
