# Supplementary material for: A rapid mixed-methods assessment of Libya’s primary care system
Source: BMC Health Serv Res. 2024 Jun 11;24:721. doi: 10.1186/s12913-024-11121-w (PMC11167861; doi:10.1186/s12913-024-11121-w)
Supplement: Supplementary file 1 — Supplementary Material 1. [file 12913_2024_11121_MOESM1_ESM.docx]

# Appendix: Topic Guide

Interviews to be conducted via Microsoft Teams

Stems are based on the PHCPI Framework

A1.1

Who generates PHC policy?

How is it disseminated?

A1.B

Is there a national quality policy, or a national quality strategy?

A2.A

Please describe the payment system for PHC workers

How are funds allocated?

What do you see as the main challenges with the current methods of allocating funds and payment models for PHC and the wider care pathway in relation to the design and delivery of models of integrated care?

A3.A

How well does the national Health Information System work in terms of ongoing, systematic collection, analysis, and interpretation of health-related data essential to the planning, implementation, and evaluation of public health practice?

Does the government have routine surveillance systems for narrow conditions and populations?

Are there any examples of good practice in relation to the use of data for population health needs assessment, risk profiling, health atlas mapping etc.?

C2.C

Are there systems in place to routinely collect health and facility data. These are fed up to the national level, with interim analyses occurring at the facility/regional levels as well.

Which data are collected?

How are they used?

A3.B

Is there a standardised priority setting approach that involves assessing existing and emerging health needs (see A3.a Surveillance), stakeholder engagement and social accountability, use of an explicit process, consideration of values and context, funding programs, communicating decisions, and managing feedback and demands from stakeholders at national and sub-national levels - at the national and regional levels

A3.C

Are there any established systems to routinely incorporate new evidence from research, or reviewing lessons learned?

Is this true: “There is a lot of innovation, with new models of care being trialled by various different partners, and the establishment of the PHC Institute. This offers scope to establish learning mechanisms.”

B2

Do you have a map of all PHC facilities?

Can you provide an overview of the state of PHC facility infrastructure

B3

How do health management information systems work across the country?

How many different systems are there? Are they interoperable?

What proportion of facilities use paper vs EHRs?

Are any routine analyses run on health data? At the local level? By whom?

C1

What processes are used to gather local intelligence on the risk factors influencing the health of local populations?

What processes are used to analyse and interpret these data?

What processes are used to develop approaches and actions to address the unique needs of local populations?

B4

What proportion of PHC facilities are staffed by a doctor or clinical officer equivalent with PC training?

Check: “Overall, all districts have the requisite number of health professionals, however they tend to cluster around urban centres leaving rural areas underserved.”

There are also 302 facilities that do not offer any services yet collectively employ 14,500 health workers - please explain how this happens.

Do you have any data on the % of population that lacks access to PC? Or map of clinician density?

C1.A

To what extent is there local priority setting (this entails the translation of national or regional policies into local strategic action plans that respond to the burden of disease and needs and preferences of the population).

C1.B

To what extent is there community engagement (a process of developing relationships that enable stakeholders to work together to address health-related issues and promote well-being to achieve positive health impact and outcomes. The definition is guided by these caveats: 1) stakeholders comprise multiple communities that could include community members, patients, health professionals, policy-makers and other sectors; 2) desired relationships are characterized by respect, trust and a sense of purpose; 3) health-related issues include public health events such as emergencies.)

Do you have any good examples?

Are any of the PC pilots including community engagement mechanisms?

C1.C

What are the main barriers for introducing empanelment?

A decent proportion of patients are asked to ‘register’ on arrival, according to the WB survey - what does this entail and how are the data used?

C1.D

Do you have any non-migrant examples of proactive population outreach (involves health systems actively reaching out to communities, particularly those that are underserved or marginalized, to provide necessary services aligned with local priorities and burden of disease, and link those in need to primary health care. Examples of proactive population outreach interventions include mobile health units, transport systems, home based care, telemedicine and proactive follow-up with patients with chronic illness.)?

C2.B

Can you speak to facility management capability (includes the technical training and skills of facility managers to effectively organize facility operations, motivate staff, manage budgets and deploy resources, and react to new challenges.)

C2. D

Please speak to performance measurement and management (includes both supportive and continuous supervision of staff as well as the routine establishment of performance targets, monitoring of progress towards these targets, and implementation of quality improvement initiatives to address identified gaps.)

Are there examples of public transparency in performance measurement whereby local populations can hold health care providers accountable for quality and efficiency?

C3.A

All government PHC is free at the point of use, but user fees are sometimes levied. Please speak to this.

C3.B

Do you have data on mean distance to the closest facility?

Physician/PHC density data?

Check: There are no standards for minimum distances (in time or km).

C3.C

Check: The majority of PHC facilities use walk-in systems - patients show up and wait for services without an appointment. This can lead to queues at busy times.

C4.A

How many family medicine trained doctors are there in Libya?

What is the aspiration?

C4.B

To what extent is this true: “Family medicine is recognized by the Libyan Board of Medical Specialties, however there is a dearth of trained family physicians working in Libya. Furthermore, there is no agreement as to the job description of doctors, nurses and other clinical team members providing care at PHC facilities. This makes it hard to guarantee that a trained provider with the requisite skills will be present”

C4.C

What motivates PHC staff?

How much of a role does money play?

C4.D/E

Do you have any data on patent safety? Or patent safety policies or mechanisms? Or preventable harm?

Do you have any data on trust in PHC?

C5.A

Do you have any data on first-contact care-seeking? Or reasons for bypassing PHC?

To what extent is primary care a “gatekeeper” for secondary care?

C5/B

I’d like to hear clinicians talk about barriers and enablers for providing continuity of care, including note-making, relational continuity, EMR use, and general use of pre-existing medical records.

C5.C

How comprehensive are the services offered? Preventive to rehabilitative, and in terms of number of different services

Can first-level PHC manage the majority of presenting complaints without referral?

To what extent is care person-centred, going beyond consideration of biology?

C5.D

If not already covered, please explain coordination across time and levels of care, as well as informational continuity

C5.E

Does med training include person-centered care?

Please can you speak to the culture of shared decision-making

**Additional IPCHS questions:**

***Strengthening Governance and Accountability***

Participatory governance and mutual accountability are important features of IPCHS.

· What would you say are the main challenges facing Libya in relation to building participatory governance and mutual accountability for health systems locally?

Re-orienting the model of care

· What is the state of current practice in terms of:

- multi-disciplinary team working in primary care?
- primary care as a “gatekeeper” to secondary care?
- Use of technology?

Care-coordination

· What is the state of current practice in terms of:

- Care coordination for individuals?
- Provider collaboration for care coordination along a programme?
- Cross-sector coordination?

Enabling Environment

· What are the main issues and challenges facing Libya in relation to

- Health workforce generic skills and capabilities (understanding of population health, health systems, health financing, payment models, multi-disciplinary team working, care pathway design, etc)
- Regulatory frameworks to support integration

What do you see as the main challenges facing patients and providers?

What are efforts to advance primary care being focused at the moment?

Which areas do you think represent priority areas for reform?
